# Local Graft Irradiation for Kidney Allograft Rejection: A Case Series and Review of the Literature

**DOI:** 10.5812/numonthly.16262

**Published:** 2014-04-28

**Authors:** Mohammad Kazem Fallahzadeh, Sarah Khan, Gazi B. Zibari, Sandeep Patil, Neeraj Singh

**Affiliations:** 1 John C. McDonald Regional Transplant Center, Willis Knighton Health System, Shreveport, USA; 2 Division of Nephrology, Department of Medicine, LSUHSC-S, Shreveport, USA

**Keywords:** Rejection, Graft, Immunosuppression, Kidney, Radiotherapy, Transplantation

## Abstract

**Introduction:**

Due to its immunosuppressive properties, local graft irradiation (LGI) has been proposed as a second line therapy for treatment of acute kidney rejection.

**Case Presentation:**

In this case-series we report 6 patients with biopsy proven acute kidney allograft rejection refractory to conventional antirejection therapy who underwent LGI for treatment of acute rejection at our center. Three of these patients had living donor transplants, 2 had deceased donor transplants, and one had received a simultaneous kidney/pancreas transplant. All patients were treated with anti thymocyte-globulin or muromonab-CD3, and intravenous steroids for initial treatment of rejection. Three patients also received intravenous immunoglobulin. LGI was tried as a last resort and was well tolerated and resulted in either improvement or stabilization of renal function in 5 patients. One patient could not be given the complete course of chemical immunosuppression for treatment of rejection due to concomitant cryptococcal meningitis and was switched to LGI with good short-term response.

**Discussion:**

Our results suggest that LGI could be considered a second line therapy to the conventional anti-rejection therapy for patients with refractory acute kidney allograft rejection, or for patients who cannot receive systemic immunosuppression due to severe infection.

## 1. Introduction

Over the last 40 years, kidney transplantation has become the preferred method of treatment for end-stage renal disease (ESRD). Acute rejection is the most important cause of kidney allograft failure. The conventional anti-rejection therapy includes intravenous steroids and antithymocyte globulin (ATG) (1, 2). In cases of antibody mediated rejection, therapies like plasmapheresis, intravenous imunoglobulin (IVIG), rituximab and/or bortezomib are often added (1-3). Local graft irradiation (LGI) is a lesser known therapy for the treatment of rejection. The immunosuppressive properties of the radiation therapy have been known for a long time. These properties are currently harnessed to eliminate lymphocytes in blood transfusion products in order to prevent transfusion associated complications like graft versus host disease (4). Radiation therapy has also been used in the field of renal transplantation since its early days (5). Initial studies showed improved graft survival with different methods of radiation such as extracorporeal blood irradiation (6), total lymphoid irradiation (7) and local graft irradiation (LGI) (8). Despite the initial promising results in small retrospective studies (8), later randomized trials did not show the beneficial effects of LGI for treatment of acute allograft rejection as first line adjunct therapy added to a conventional anti-rejection regimen like antithymocyte globulin (ATG) and steroids (9-11). However, the efficacy of LGI as a second line therapy after failure of conventional antirejection treatment has been more successful (12-17). In this study, we have reported our experience with 6 patients with acute kidney allograft rejection who were treated with LGI as a second line therapy. In addition, we have reviewed the previous literature on the use of LGI for acute kidney allograft rejection. This study was approved by Institutional Review Board of our institution and was done in accordance with the Helsinki Declaration of 1975 (as revised in 1983). In this study the charts of all patients with acute kidney allograft rejection who were treated with LGI at our center were retrospectively reviewed. Rejection refractory to medical therapy was defined as an acute rejection that did not respond to conventional anti-rejection therapy (antithymocyte globulin [ATG] or muromonab-CD3 per physician’s discretion in addition to intravenous steroids) with failure of serum creatinine to trend down until at least 1 week after the completion of anti-rejection therapy. IVIG was also given to patients with positive donor specific antibody (DSA) levels which were defined as a mean fluorescence intensity ≥ 2000. The DSA level were measured by a Luminex analyzer (Luminex Corporation, Austin, TX, USA). Episodes of acute rejection were confirmed by renal biopsy. The dose range of LGI treatment varied from 600–800 cGy given in the fractions of 150 cGy to 200 cGy daily for 4 days using either 6MV and/or 18MV photons.

## 2. Case Presentation

The patients’ demographics, clinical characteristics, radiotherapy doses and final outcomes are shown in Table 1. Per our center’s protocol all patients except patient No 4 received 7-10 doses of ATG (1.5 mg/kg IV daily) or 10 doses of muromonab-CD3 (5 mg IV daily) along with high dose IV steroids for treatment of acute allograft rejection. Except for patient No 6, the kidney function improved temporarily in all patients who were treated with LGI. Our patients’ kidney allograft survival at 1, 3, 6 and 12 months was 83%, 83%, 33% and 17%, respectively. Patient No 4 was diagnosed with cryptococcal meningitis 3 days after biopsy proven allograft rejection, and therefore he was treated with IV steroids and ATG for only 3 days. He was switched to LGI on day 5 with good response.

**Table 1.** The Demographics, Clinical Characteristics, Radiotherapy Doses and Final Outcomes of Patients With Acute Kidney Allograft Rejection Refractory to Medical Therapy Treated With LGI ^a^

Patients No.	1	2	3	4	5	6
**Age at time of transplantation, y**	28	26	21	38	32	35
**Type of kidney transplantation **	Deceased donor	Living donor	Living donor	Deceased donor (simultaneous kidney/pancreas transplant)	Living donor	Deceased donor
**Etiology of kidney disease**	Hypertensive nephropathy	IgA nephropathy	Reflux nephropathy	Diabetic nephropathy	Diabetic nephropathy	Hypertensive nephropathy
**Baseline serum creatinine range, mg/dL**	2.0-2.4	1.5-2.1	2.5-3	2.5-2.7	1.5-2.1	2.5-3.2
**Time from kidney transplantation to rejection, mo**	54	22	36	29	75	21
**Allograft kidney biopsy**	Grade 1A rejection, Peritubular capillaritis, C4d+, No fibrosis	Grade 1B rejection, Peritubular capillaritis, C4d+, No fibrosis	Grade 1B rejection, Thrombotic microangiopathy, C4d+, Minimal fibrosis	Grade 1B rejection, C4d-, Moderate fibrosis	Grade 1A rejection, C4d-, Moderate fibrosis	Grade 1B rejection, Peritubular capillaritis, C4d-, No fibrosis
**DSA**	+	-	+	-	-	+
**Medical Management**	ATG (1.5 mg/kg × 8 doses), IVIG (50 g × 1 dose), IV steroids taper	Muromonab-CD3 (5 mg × 10 doses), IV steroids taper	ATG (1.5 mg/kg × 10 doses), IVIG (40 g × 1 dose), IV steroids taper	ATG (1.5 mg/kg × 3 doses), IV steroids taper	ATG (1.5 mg/kg × 7 doses), IV steroids taper	ATG (1.5 mg/kg × 10 doses), IVIG (40 g × 2 doses), IV steroids taper
**Maintenance therapy before rejection**	Mycophenolic acid 720 mg BID, tacrolimus 16 mg BID, prednisone 2.5 mg QD	Mycophenolate mofetil 1000 mg BID, tacrolimus 2 mg BID, prednisone 2.5 mg QD	Mycophenolate mofetil 1000 mg BID, tacrolimus 2 mg BID, prednisone 20 mg QD	Sirolimus 1 mg QD, tacrolimus 1 mg BID, prednisone 20 mg QD	Mycophenolate mofetil 1000 mg BID, tacrolimus 2 mg BID, prednisone 10 mg QD	Mycophenolate mofetil 750 mg BID, tacrolimus 3 mg BID, prednisone 2.5 mg QD
**Prior Rejections **	4	3	3	6	4	0
**Time difference between biopsy and radiotherapy, d**	15	17	20	5	16	18
**LGI Dose**	200 cGy/d × 4 using 6 MV/18 MV photons	150 cGy/d × 4 using 18 MV photons	150 cGy/d × 4 using 6 MV photons	200 cGy/d × 4 using 6 MV/18 MV photons	200 cGy/d × 4 using 6 MV/18 MV photons	200 cGy/d × 4 doses using 6 MV photons
**Change in Serum creatinine, mg/dL within a month post-LGI**	Serum Cr decreased from 5.2 mg/dL to 4.4 mg/dL a month after radiotherapy	Serum Cr decreased from 4.3 mg/dL to 3.3 mg/dL at 2 weeks	Serum Cr decreased from 5.9 mg/dL to 4.8 mg/dL at 2 weeks	Serum Cr decreased from 6.9 mg/dL to 3.8 mg/dL at 3 weeks	Serum Cr remained stable between 3.9-4.1 mg/dL.	Serum Cr increased from 7.0 mg/dL to 7.7 mg/dL a week after LGI
**Outcome post LGI**	Renal function has remained stable since LGI	Renal function stabilized for 2 months but deteriorated at 3 months post-LGI and patient initiated dialysis	Renal function stabilized for 6 months but deteriorated at 7 months post LGI and patient initiated dialysis	Renal function stabilized for 2 months but deteriorated at 3 months post LGI and patient initiated dialysis	Renal function stabilized for 3 months and but deteriorated at 4 months post- LGI, patient initiated dialysis	Renal function did not improve post-LGI therapy and patient initiated dialysis 1 week post-LGI
**Graft survival, post-LGI, months**	Kidney allograft functional till last follow-up of 24 months	3	7	3	4	0

^a^ Abbreviations: ATG, antithymocyte globulin; Cr, creatinine; DSA, donor specific antibody; LGI, local graft irradiation

## 3. Discussion

Our results suggest that for patients with refractory acute kidney allograft rejection LGI may be considered as a second line therapy to conventional antirejection therapy including intravenous steroids and ATG. In our study, 5 out of 6 patients with refractory rejection responded favorably to LGI. In addition, patient No.4 had concomitant acute rejection and cryptococcal meningitis and responded well to LGI suggesting that LGI may also be indicated for patients with acute rejection when systemic immunosuppression is contraindicated as in the setting of superimposed serious systemic infection. In our study, LGI was found to be safe and without any major adverse effect. Although the initial reports on using LGI as an adjuvant therapy for acute rejection were encouraging (8), 3 later randomized trials did not show the efficacy of LGI as an addition to the chemical immunosuppressive therapies in the first line of treatment for acute kidney rejection (9-11). Later studies showed that LGI could be used as a second line therapy when the acute kidney rejection failed to respond to medical immunosuppressive therapy (Table 2). In 1984, Halperin et al. (12) demonstrated that in 53 patients with acute allograft rejection resistant to medical therapy, radiotherapy resulted in improvement or stabilization of renal function in 42% of the patients a month after LGI. But one year post-LGI, only 21% of these allografts were still functional. In this study, 10 patients with acute allograft rejection in whom immunosuppressive therapy was contraindicated because of either systemic infections or hematologic dyscrasias received LGI; 1 month and 1 year graft survival rates in this group were 90% and 40%, respectively. Later, Noyes et al. (14) and Nuyttens et al. (15) reported a 1 year graft survival of 49% and 50%, respectively, in patients who had acute rejection refractory to medical therapy and who received LGI. In a study by Chen et al. (17) which mostly included patients with acute allograft rejection resistant to medical therapy, 1 year graft survival rate was 60%. Wahl et al. (16) reported a 1 month graft survival of 63%, but the 1 year graft survival was poor at 31%. Similar to previous reports, our study also shows a good short term graft survival (83% at 1 and 3 months) but poor long term graft survival (17% at 1 year) when LGI is given as a second line agent for refractory rejection.

The mechanism of action of LGI in the treatment of rejection appears to be the elimination of lymphocytes. Dividing cells like lymphocytes, both in circulation and in tissues, have been shown to be particularly sensitive to radiation therapy (18). Radiation can directly damage DNA; however, generation of free reactive oxygen species from the radiolysis of water by radiation is the most important cause of DNA damage and cell death. The T-cells involved in acute cellular rejection are located in the transplanted kidney, but plasma cells that produce antibodies involved in antibody mediated rejection are mostly located in the lymph nodes and bone marrow (19). Therefore, by preferentially targeting T-cells located in the kidney, LGI is probably more effective against acute cellular rejection compared with antibody mediated rejection. Compared with sensitive dividing cells like lymphocytes, non-dividing cells like renal cells are less sensitive to radiation therapy (18). Moreover, the dose of radiation in LGI is low. Therefore, LGI appears to be safe in the treatment of acute kidney allograft rejection without major side effects like radiation nephritis (10, 16).

LGI may be tried as a second line therapy for treatment of kidney allograft rejection refractory to conventional anti-rejection medical therapy. In addition, LGI may be a useful modality for treatment of kidney allograft rejection when systemic immunosuppression is contraindicated due to concomitant severe and serious infection. The benefits of LGI should be confirmed in future prospective randomized controlled trials.

**Table 2.** Summary of Studies Reporting the Use of LGI as a Second Line of Therapy for Acute Kidney Allograft Rejection ^a, b^

Author	Number of Patients	Selection Criteria	Dose Range, cGy	Median Dose, cGy	One Month Graft Survival Post-LGI, %	One Year Graft Survival Post-LGI, %
**Halperin et al. (12)**	53	Refractory acute allograft rejection	300-1200	600	42	21
	10	Acute allograft rejection with contraindicated immunosuppressive therapy due to systemic infection or hematologic dyscrasias	300-1200	600	90	40
**Jagetia et al. (13)**	6	Refractory acute allograft rejection	450-600	450	50	17
**Noyes et al. (14)**	72	Refractory acute allograft rejection	800	800	NA	49
**Chen et al. (17)**	53	Refractory acute allograft rejection (75 % of patients)	600	600	83	60
**Nuyttens et al. (15)**	20	Refractory acute allograft rejection	450	450	NA	50
**Wahl et al. (16)**	33	Refractory acute allograft rejection	800	800	63	31
**Current study **	6	Refractory acute allograft rejection	800–600	733	83	17

^a^ Abbreviations: LGI, local graft irradiation; NA, not available.

^b^ Biopsy proven acute allograft kidney rejection resistant to medical therapy.
